# Gradual step-up weaning improves liberation in chronically ventilated tracheostomized patients

**DOI:** 10.62675/2965-2774.20260255

**Published:** 2026-03-16

**Authors:** Caroline Colombo, Cassiano Teixeira, Sílvia Regina Rios Vieira

**Affiliations:** 1 Hospital Moinhos de Vento Intensive Care Unit Porto Alegre RS Brazil Intensive Care Unit, Hospital Moinhos de Vento - Porto Alegre (RS), Brazil.; 2 Universidade Federal do Rio Grande do Sul Hospital de Clínicas de Porto Alegre Intensive Care Unit Porto Alegre RS Brazil Intensive Care Unit, Hospital de Clínicas de Porto Alegre, Universidade Federal do Rio Grande do Sul - Porto Alegre (RS), Brazil.

Patients requiring prolonged mechanical ventilation (MV ≥ 14 days) and tracheostomy pose significant clinical and logistical challenges in the intensive care unit (ICU).^(1-5)^ Given the limitations of conventional spontaneous breathing trial (SBT) strategies in this population, we evaluated whether a structured, gradual step-up SBT protocol could improve ventilator liberation among tracheostomized patients with chronic respiratory failure.

We conducted a retrospective quasi-experimental study in a tertiary ICU in Brazil, comparing two distinct periods: 2015 - 2016, during which weaning was based on prolonged, continuous T-tube SBT conducted by the ICU team (prolonged SBT group); and 2018 - 2019, when a time-structured, incremental weaning strategy was adopted (gradual step-up SBT group). Both groups received the same standard of care, including respiratory and peripheral muscle training, tracheostomy management, and speech-language therapy. At the time of SBT initiation, all patients had a RASS between +1 and –1, were on pressure-support ventilation ≤ 18cmH_2_O, positive end-expiratory pressure (PEEP) ≤ 8cmH_2_O, and fraction of inspired oxygen (FiO_2_) ≤ 50%.

A total of 114 adult tracheostomized patients who had been mechanically ventilated for more than 14 days were included. The primary outcome was successful weaning, defined as remaining off MV for more than 5 consecutive days. In the prolonged SBT group (n = 63), patients underwent extended SBTs without a structured progression. The protocol resembled the approach described by Jubran et al.:^(6)^ on Day 1, patients were allowed to breathe unassisted for up to 12 hours, followed by 12 hours of assist-control ventilation. From Day 2 onward, patients were disconnected from the ventilator and permitted to breathe spontaneously via tracheostomy for up to 24 hours daily. In contrast, the gradual step-up group (n = 51) followed a defined protocol with daily increases in SBT duration, starting at 2 hours and increasing by 2 - 4 hours each day until reaching 24 hours. Both groups had similar baseline characteristics, including age (72.5 ± 15.7 *versus* 69.1 ± 19.3 years; p = 0.07), Simplified Acute Physiology Score (SAPS) III scores (55.7 ± 10.7 *versus* 56.1 ± 12.3; p = 0.06), frailty index (2.4 ± 0.5 *versus* 2.5 ± 0.3; p = 0.13), and MV duration prior to tracheostomy (17 [14 - 25] *versus* 16 [14 - 21] days; p = 0.07). Although comorbidities were more frequent in the step-up group (≥ 2 comorbidities: 58.8% *versus* 28.6%; p < 0.003), no differences were observed in the prevalence of reduced ejection fraction heart failure or chronic obstructive pulmonary disease.

Overall, 55% (63/114) of patients were successfully weaned. The success rate was significantly higher in the step-up group compared to the prolonged SBT group (70.6% [36/51] *versus* 43.0% [27/63]; p = 0.005). Kaplan-Meier analysis confirmed a greater probability of ventilator liberation in the step-up group (log-rank p = 0.003). After adjusting for age, sex, ICU admission diagnosis, SAPS III, Charlson comorbidity index, frailty, and MV duration prior to tracheostomy, the gradual weaning strategy remained independently associated with weaning success (hazart ratio [HR] 2.15; 95%CI 1.02 - 4.53; p = 0.04) (Figure 1).

**Figure 1:**
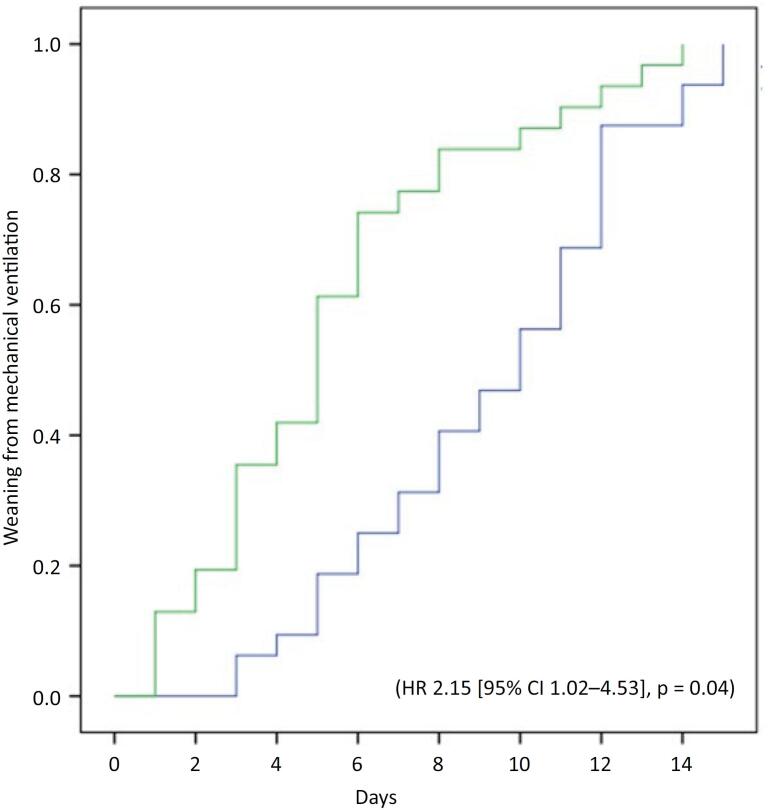
Cumulative probability of successful weaning according to weaning strategy.

HR - hazard ratio; 95%CI - 95% confidence interval.

Cumulative probability of successful weaning from mechanical ventilation among tracheostomized patients using prolonged spontaneous breathing trial (blue line) and gradual step-up spontaneous breathing trial (green line) strategies. Adjusted HR 2.15 [95%CI, 1.02 - 4.53]; p = 0.04.

Notably, the gradual step-up protocol yielded better outcomes even in a cohort with a greater burden of comorbidities. This suggests the approach may be better suited to the limited diaphragmatic reserve and impaired ventilatory drive commonly seen in patients with chronic respiratory failure.^(6-8)^ The time-structured progression likely reduces respiratory muscle fatigue while facilitating physiological adaptation to unassisted breathing. This study has several limitations. Its retrospective, single-center design and lack of long-term follow-up limit the generalizability of the findings. Standardized assessments of respiratory muscle strength were not performed, and relevant clinical variables known to affect weaning outcomes - such as incidence of delirium, occurrence of nosocomial infections, and sedation strategies - were not systematically analyzed. Furthermore, other complications associated with prolonged ICU stay, which could also contribute to weaning failure, were not captured in the dataset. However, no significant changes in ICU staffing, protocols, or technology occurred between the two study periods, minimizing potential secular bias.^(9,10)^

In conclusion, our findings support the use of structured, stepwise SBT protocols in ICUs caring for chronically ventilated, tracheostomized patients. A gradual approach to weaning was associated with higher rates of ventilator liberation, even among those with greater comorbidity burden.

## Data Availability

The contents underlying the research text are included in the manuscript.
